# Participation in a randomised controlled trial (RCT) of metformin in gestational diabetes mellitus (GDM): pregnant women’s perceptions and experiences of the decision-making process

**DOI:** 10.12688/hrbopenres.13289.1

**Published:** 2021-06-14

**Authors:** Sinead Wallace, Catherine Houghton, Fidelma Dunne

**Affiliations:** 1 HRB Clinical Research Facility, National University of Ireland Galway, Galway, Ireland; 2 School of Nursing and Midwifery, Áras Moyola, National University of Ireland Galway, Galway, Ireland; 3 School of Medicine, National University of Ireland Galway, Galway, Ireland

**Keywords:** Randomised controlled trial, Pregnancy, Gestational diabetes mellitus

## Abstract

**Background:** Research in pregnancy and childbirth is required to advance healthcare needs for this population. Fears around potential fetal risk and the history of drug scandals renders it an area of research that is somewhat neglected. Due to the growing medical complexities facing pregnant women, efforts have been made in recent times to recognise the ethical importance of including this population in clinical research. Although clinical trials are becoming more common in pregnancy, recruitment of this population remains difficult with a common assumption that pregnant women would be reluctant to participate in clinical trials. This study set out to explore pregnant women’s perspectives and experiences of the decision-making process to participate in a randomised controlled trial of metformin in gestational diabetes mellitus (the EMERGE clinical trial).

**Methods:** This study employed a qualitative descriptive design with thematic analysis. Data were collected by conducting individual semi-structured interviews (n=11) with participants (n=9) and decliners (n=2) of the EMERGE clinical trial.

**Results: **The main findings reveal that a significant perception of personal benefit from participation was the biggest influence on women’s decisions to participate. Concerns about the impact of gestational diabetes on their pregnancies, the option of a favourable intervention treatment, a low perception of risk associated with the trial and the opportunity to help medical research appeared to have significantly influenced their decision. Receiving detailed information, personal interactions with the study team, a perception of voluntariness in participation and accessibility of the trial positively impacted on women’s decisions to participate.

**Conclusions:** Personal contact during recruitment, presenting clear and thorough trial information, providing previous participant testimonials, and facilitating women to participate in clinical trials are all important strategies when trying to enhance recruitment in pregnancy trials. Further research on pregnant women declining participation in clinical trials is needed.

## Introduction

Historically, pregnant women were excluded from research as they were thought of as a vulnerable population in need of protecting from the dangers of research ( Ballantyne *et al.*, 2017; Matsui, 2015). Pregnancy was often deemed an automatic exclusion criterion for clinical trials ( Matsui, 2015). Main reasons for their exclusion were often related to the classification of pregnant women as a vulnerable population, fear of causing harm to the fetus and doubts whether pregnant women would participate in clinical research ( Blehar *et al.*, 2013). Concerns about lack of evidence regarding safety and unknown risks to the fetus are often reasons why drug companies, institutional review boards and researchers remain reluctant to include pregnant women in clinical trials ( Allesee & Gallagher, 2011; Matsui, 2015). However, reluctance to treat pregnant women because of this carries its own risks ( Foulkes *et al.*, 2011). The risk of untreated or mistreated conditions in pregnancy due to fears about fetal risk are certainly worth considering, therefore, the risks of not being included in clinical research, for both the pregnant woman and her baby, should also be taken into account ( Hunt *et al.*, 2017).

It appears the age-old focus on the overriding concern of protection of the fetus is being re-evaluated ( Foulkes *et al.*, 2011). A meeting of the global forum on bioethics in research in 2016, highlighted the need for responsible inclusion of pregnant women in research unless a valid reason for exclusion exists and that the assessment of risks should include the risks of pregnant women not being included in research ( Hunt *et al.*, 2017).

Research in pregnancy is needed to advance and inform healthcare decisions and treatment options for this population ( Foulkes *et al.*, 2011; Frew *et al.*, 2014). Given the history and reluctance to conduct clinical research in pregnancy, it is not surprising that a recent survey of registered clinical trials of pharmacological interventions in pregnancy by Scaffidi *et al.* (2017) showed that only 0.32% of all active registered studies were pregnancy drug trials. More evidence to guide clinical decision making in pregnancy is needed ( Domínguez *et al.*, 2012). Furthermore, as there are matters specific to pregnancy that must be considered, the importance of conducting research in the pregnant population cannot be underestimated.

As the provision of evidence based, safe and effective treatment during pregnancy is required ( Matsui, 2015), the importance of including this population in clinical research must be recognised so that ultimately knowledge can be gained to help ensure healthy mothers and babies during and after pregnancy ( Foulkes *et al.*, 2011), and to provide adequate evidence guiding clinical care for this group ( Ballantyne *et al.*, 2017).

### The EMERGE clinical trial

A study is currently underway, and actively recruiting participants, in Ireland to evaluate the effectiveness of Early MEtformin in addition to usual care in the Reduction of Gestational diabetes mellitus Effects, the EMERGE clinical trial. This is a randomised double-blind placebo-controlled trial which is inclusive of women diagnosed with gestational diabetes mellitus (GDM) and involves evaluating the use of metformin, in addition to standard care, at the time of diagnosing GDM with the aim of providing evidence to support early active management with metformin in women with GDM in the Irish population. Women diagnosed with GDM, who fulfil the eligibility criteria for inclusion, are invited to participate in this clinical trial during their pregnancy. Further information on the EMERGE clinical trial can be found at https://clinicaltrials.gov/ct2/show/NCT02980276.

Since the re-evaluation of some of the ethical issues surrounding pregnancy research, there has been a call to encourage drug trials in pregnancy, however, there still appears to be little progress in that area ( Endicott & Haas, 2012). Not alone is there a relatively low number of trials taking place during pregnancy ( Domínguez *et al.*, 2012), but often some that are conducted face significant challenges with recruitment ( Endicott & Haas, 2012; Strömmer *et al.*, 2018). Challenges in recruitment can lead to unrepresentative samples, the validity of the trial may be jeopardised, study costs may increase due to extension and overall staff morale may be affected ( Strömmer *et al.*, 2018). Enrolment into randomised trials in pregnancy remains challenging ( Oude Rengerink *et al.*, 2015) and personal factors are often cited as influencing participation in clinical trials. There also appears to be a common assumption that perhaps pregnant women would be reluctant to participate in clinical research ( Ballantyne *et al.*, 2017; Frew *et al.*, 2014).

In general, studies on women’s views and experiences of participation, or being approached to participate, in pregnancy trials appears to be somewhat limited. Some studies have examined women’s opinions of research in pregnancy in general. Some were based on pregnant women having, or being at risk of developing, a particular condition in pregnancy and others were based on healthy volunteers and hypothetical participation in pregnancy research. In relation to specific obstetric conditions it appears that there are only a handful of studies that have explored women’s decisions to participate in an IMP (investigational medicinal product) clinical trial. The QUOTE study ( Smyth *et al.*, 2012) explored the decision-making process for women entering a randomised controlled trial (RCT) of prophylactic anticonvulsants for women with severe pre-eclampsia (the Magpie trial). Kenyon *et al.* (2006) explored women’s experiences of being recruited to ORACLE, an RCT of antibiotics in pre-term labour and Mohanna & Tunna (1999) looked at reasons for declining participation in a clinical trial to assess the efficacy of nifedipine in preventing the onset of labour before 37 weeks’ gestation in a high-risk group (the PLANET trial). Oude Rengerink *et al.* (2015) also looked at barriers and motivators to participation across a range of clinical trials in pregnancy (mostly related to pre-term labour/birth and hypertension).

To advance our knowledge and insight into women’s views on clinical trials, in particular in relation to GDM, it is certainly worthwhile exploring their opinions. This study aimed to explore personal perspectives of pregnant women and factors that influence participation in clinical drug trials in pregnancy, with a particular focus on the decision-making process to enter the trial.

The specific study objectives were:

1. To explore the decision-making process for women with GDM to participate in the EMERGE clinical trial.
2. To explore the factors influencing their decision to participate.
3. To explore differences in the perceptions of women who decline participation with women who agree to participate.

## Methods

### Study design

A qualitative descriptive design was chosen to best capture the study objectives. This design allows a clear description of a specific experience from the perspective of the experiencing individual ( Magilvy & Thomas, 2009). It is useful to obtain straight answers to research questions that may be looking at people’s responses to an event or experience; concerns they may have about an event; or reasons why they would or would not use a service ( Sandelowski, 2000).

### Participants and setting

This study was conducted in the Health Research Board (HRB) Clinical Research Facility, University Hospital Galway. Pregnant women who had a positive diagnosis of GDM up to 28 weeks and 6 days gestation, with a singleton pregnancy were invited to participate in the EMERGE clinical trial. Table 1 provides a full account of the eligibility criteria for the EMERGE clinical trial. Women invited to participate in the EMERGE clinical trial were eligible to participate in this study. Participants were approached and recruited for this study between May and July 2018.

**Table 1.** Overview of eligibility criteria for EMERGE clinical trial.

Inclusion Criteria
Participants between 18–50 years old with a singleton pregnancy of gestation up to 28 weeks (+6 days) Positive diagnosis of Gestational diabetes mellitus according to International Association of Diabetes and Pregnancy Study Groups (IADPSG) criteria
Exclusion criteria
Participants who have an established diagnosis of diabetes (Type 1, Type 2, monogenic or secondary) Participants with a fasting glucose level of ≥ 7 mmol/l or a 2-hour value ≥ 11.1 mmol/L Known intolerance or contraindication to the use of metformin Major congenital malformations or known small for gestational age fetus Current gestational hypertension, pre-eclampsia or ruptured membranes Significant gastrointestinal problems, history of drug/alcohol use, serious mental health issues with potential to affect compliance with trial Congestive heart failure Rare hereditary problems of galactose intolerance, Lapp lactose deficiency or glucose-galactose malabsorption

A pre-determined sample size was not planned for this study. In general, qualitative research does not require a specific sample size calculation prior to commencing a study and sample size is often much smaller than in quantitative research. The aim of this study was narrow, seeking specificity to the topic of interest and a strong dialogue, so a larger sample size was not deemed necessary ( Malterud *et al.*, 2016). A purposeful approach to sampling was adopted, with the aim of selecting “information rich” cases to provide the greatest insight ( Devers & Frankel, 2000). Typically, it can range anywhere from three up to twenty participants who have experienced the phenomenon of interest, can communicate with the researcher and are willing to talk about their experiences ( Magilvy & Thomas, 2009). Within this approach, a maximum variation sampling technique was adopted to obtain a broad range of information-rich cases for the purpose of the study. Thus, sampling of a range of demographics such as age, parity (number of births > 24 weeks gestation) and acceptors and decliners of the EMERGE trial were incorporated to seek a variation in perspectives on the topic of interest. The aim was to achieve “data adequacy”, which refers to quality, sufficiency and richness of the data in the context of the research question ( Levitt *et al.*, 2017) In total, eleven participants (nine participants and two decliners of EMERGE) were included in this study.

Eligible participants who were already enrolled in the EMERGE clinical trial were informed about this study, and invited to participate, by the researcher for this study (who was also a member of the EMERGE clinical trial team). Eligible participants who had declined participation in EMERGE were informed about this study at the point of contact with the EMERGE study team where they declined participation in EMERGE. Information about the study was provided in the form of a participant information leaflet with additional information provided in discussion with potential participants by the study researcher. Participants were given time to decide whether they wanted to participate and follow up occurred after a few days of receiving the information to allow sufficient time to make a decision. With those agreeable to participate, a convenient time, suiting both the participant and the researcher, was arranged to conduct the interview.

### Ethics

Ethical approval to conduct this study was obtained from the Clinical Research Ethics Committee, Merlin Park University Hospital, Galway, Ireland. Reference No: C.A. 1958, dated 29 ^th^ May 2018. All participants provided written and verbal informed consent to participate in this study.

### Data collection

Data were collected by the main researcher (SW) by conducting face to face individual semi-structured interviews with participants between May and July 2018 in which the EMERGE trial was concurrently running. Semi- structured interviews allow for flexibility and probing into topics areas of interest while still maintaining the direction of the interview to answer the research questions proposed ( Alshenqeeti, 2014). The interview guide ( Wallace, 2021), with open questions and probes, was developed from a review of previous literature, the study objectives and pertinent information specific to the EMERGE trial. The interviews were piloted with the first two participants in this study to assess the suitability of the interview guide and the appropriateness of the questions to ensure understanding from the participant perspective. It was apparent from the pilot interviews that there was a good understanding from the participant perspective of the interview questions, therefore, no changes were made to the interview guide following the pilots and these interviews were included in the final data.

Interviews were audio recorded and transcribed verbatim. All transcripts were cross checked for completeness. Confidentiality of participant details was maintained throughout the study. The right to withdraw from the study at any point was emphasised to each participant. Recordings and transcripts from each participant were numerically coded by participant number to maintain anonymity. Identifiable information recorded during the interview were removed from the transcripts prior to analysis. Paper copies of data related to each participant were kept in a secured drawer in the research facility until data analysis was completed. Coded transcripts were kept in a password protected file which was accessible only by the researchers involved in the study. 

### Data analysis

Data were analysed using thematic analysis by the main researcher (SW). Themes capture important aspects of data and can be useful to summarise key features of a large data set ( Braun & Clarke, 2006). A six-phase guide by Braun & Clarke (2006) guided the thematic analysis in this study. Additionally, a qualitative data analysis software programme, QSR NVivo 11, was used to manage and facilitate some phases of the data analysis. The phases involved reading and re-reading the interview transcripts to become familiar with the content and to form initial ideas about the data. Data was coded by SW Line by line coding of each transcript was performed. Different codes were grouped into potential themes. Themes were refined and the whole data set was re-read to determine whether the themes were appropriate and relevant given the data set. Further refining and naming of the final themes resulted in a final report containing three over-arching themes with eight subthemes.

### Rigor

Rigor was demonstrated in this study by incorporating a peer debriefing strategy as described by Houghton *et al.* (2013), whereby data analysis was cross checked at intervals by an experienced qualitative researcher. Use of the QSR NVivo 11 software programme and keeping records of raw data, researcher notes and transcripts provided an audit trail for the data analysis.

Other considerations throughout the study included recognition of the researcher’s role in relation to this study and the EMERGE clinical trial and the potential introduction of bias in data collection and data analysis. Social desirability bias is the tendency of participants to respond to questions with more socially desirable responses rather than reflect their own true thoughts or feelings ( Grimm, 2010). It was acknowledged that the researcher was also a study team member involved in the EMERGE clinical trial and would have been previously known to most of the participants in this study. Therefore, to avoid/minimise social desirability bias participants were asked at the beginning of the interview to respond openly and honestly when accounting their personal opinions regarding the topics discussed. Maintenance of confidentiality of information provided was also reiterated to the participants to encourage honest and open discussion throughout the interview.

## Results

Three main themes with eight subthemes were developed in relation to women’s experiences and factors influencing the decision-making process to participate in EMERGE. These are outlined in Table 2. A description of participants’ characteristics is presented in Table 3.

**Table 2.** Themes and subthemes.

Main Themes	Subthemes
Perceived benefits of participation	Perspectives of diagnosis and treatment options
	Weighing of risks versus benefits
	Contribution to medical research and altruism
The recruitment process	Influence of others
	Information about the trial
	Voluntariness
Trial considerations	Trial design
	Trial burden

**Table 3.** Participant characteristics.

Age	3 x 25-29 years 5 x 30-35 years 3 x 36-40 years
Parity	5 x nulliparous 6 x multiparous
Highest level of education	10 x complete third level 1 x complete second level
Ethnicity	10 x white Irish 1 x non-Irish white

### Perceived benefits of participation

The main findings suggest that perceived benefits of participation significantly influenced women’s decisions to participate. Women’s perspectives of their diagnosis and treatment options appeared to be one of the most influential factors for participation and relates to how women regarded their diagnosis, what the existing treatment options for women with GDM were and the potential for a more favourable treatment option.

Avoiding insulin and a focus on an alternative treatment option appeared to be the biggest motivating factor for the participants of EMERGE. There appeared to be many negative thoughts expressed regarding the use of insulin in treating GDM. Hoping to avoid or delay the initiation of insulin was a very significant response and was a main reason for participating in the trial for many women.

*"I thought I would do anything to avoid going on insulin." Participant2*

Women felt that treatment options available to them were limited in relation to GDM.

*"it kind of gave me an additional option before the administration of insulin" Participant7*

Having to inject insulin, side effects and the responsibility associated with taking it were all reasons why women wanted to avoid insulin. An oral form of medication was viewed far more favourably.

*"Because they’re easier to take than obviously probably the insulin. I wouldn’t have to inject myself" Participant1*

Both participants and decliners of EMERGE were initially similarly overwhelmed with their diagnosis. While some thought of the trial as an opportunity to gain control and an extra option for treatment of their condition, others thought of the diagnosis as enough to deal with rather than adding to an already demanding and overwhelming diagnosis and treatment regime.

Perceived potential benefits of the intervention medication and other benefits of being involved in a clinical trial, like extra support and monitoring, incentives and continuity of care within the trial appeared to be expressed more by women than perceived risks. The biggest perceived risk seemed to be the effects medication would have on the baby. Potential long-term effects and a brief mention of fear regarding previous scandals about drug trials in pregnancy were mentioned. The fact the trial medication was not a completely novel medication appeared to put women at ease. Knowing that there have been other trials done on it and that it is used in other parts of the world to treat GDM resulted in most women perceiving little or no risk with the medication.

*"I read up about metformin and stuff and I wasn’t going to just take any drug. But because it had been used before and because there are other studies done on it, I just thought it was a pretty safe bet for me and for my baby." Participant4*

*"I researched it quite well online and I suppose metformin’s used in a lot of other countries, so I didn’t really have any concerns about it." Participant6*

Many expressed feelings of potential benefit for the baby and being able to control their GDM if they were on the active medication. Others spoke about reducing their risk of developing adverse pregnancy outcomes that are often associated with GDM.

*"Yeah and definitely I have seen a benefit you know, with my weight and I think scans have improved as well with the abdominal circumference and that, you know, the size of the baby" Participant2*

Benefits of extra monitoring they would receive in the trial and the extra support and care they would receive from trial staff was also noted by women.

*"people get to know you as well, it all makes you feel very comfortable in terms of...just that you’re being really well taken care of. I’ve always felt like I could pick up the phone and ask, as well" Participant7*

Overall, it appeared that women saw more benefits in participation than any perceived risks and there were, generally, very positive thoughts and feelings about participating in the trial.

There was also an element of altruism expressed by women in that they felt that it was important to do something to help other pregnant women and even themselves in future pregnancies. Many reported feeling good about contributing to research and most considered this another benefit to taking part.

*"Not so much me personally. I just think if you could help the next woman or as I said even in my next pregnancy" Participant8*

### The recruitment process

Aspects of the recruitment process also appeared to influence participation, such as, how others had an impact on women’s decisions, the information they received; how they received it and the perception of voluntariness associated with participation.

While many women reported that the decision to participate was a personal decision, it transpired that other people would have also had some influence or involvement in the decision-making process either through conversation with the women or when providing information. These included some healthcare professionals (HCP), the study team, women's partners and others with medical backgrounds or previous experience in research.

Discussion about the trial with members of the study team, especially the consultant endocrinologist who was also principal investigator for the trial, appeared to have a significant impact on the decision-making process as referenced by all women. The ability to ask questions and seek further information or clarification about the trial from the study team were viewed as the most important aspects of this discussion with the study team.

*"I think everyone made it, in my meeting with the research nurse and the consultant Endocrinologist, they made it very clear......They went through everything with me and spent plenty of time with me explaining, so no I didn’t have any worries" Participant2*

Several women also appreciated the fact that these were in-person discussions.

*"if it was only the leaflets yes that something would be missing but you know when you have that personal contact and we were able to ask question at that time as well" Decliner2*
*"she (study nurse) wasn’t just a name on a piece of paper, she was a human, she was in front of me," Participant4*

Detailed discussions with members of the study team were also important for women when making their decision to participate in the trial.

*"literally the consultant went through it step-by-step as well, It wasn’t just sort of, you know, ‘Do you want to do it or not?". So, I was more confident."Participant1*

Involvement of the consultant endocrinologist in the trial itself was also reassuring for women. Several women reported that they would trust their HCP to prioritise their best interests and not unnecessarily put them at risk.

*"she’s the consultant, so surely it must be good if she’s part of it, rather than just having a team of researchers who are doing the study independently. I thought it was nice that she was also involved." Participant4*

Most women reported discussing the trial with their partners, however, only a small number reported making a joint decision with them regarding participation. What appeared most commonly was that partners were encouraging and supportive of women when making their decision, but that in a lot of cases the final decision was made by women themselves.

*"he was happy to support me once I was comfortable with it", Participant7*
*"I wanted to do it myself.........he just fully supported it and just trusted my judgment I guess" Participant8*

Testimonials from previous participants about their experiences was regularly highlighted as something women felt would really help with the decision-making process and was recommended by many women.

*"that influenced me a lot, I think, that their [previous participant] information, their experience was probably the biggest factor" Participant4*

Detailed and clear written information about the trial was considered important for women along with discussions with either the consultant endocrinologist (principal investigator of the study) or the study nurse. This made women more confident and happier in making their decision

*"I felt very well informed, there’s lots of information, so that I think always helped me feel at ease," Participant7*

Although it was noted by a few women that they would have liked to have known more about the intervention medication, overall, the detailed written information, supplemented by verbal information were considered the most important ways of receiving information about the trial.

Women appeared to appreciate that their decision to participate in the trial was completely voluntary. It was referenced by many women that they never felt pressured into participating. Comments from a decliner recognised that saying no would not be easy and that there may have been some potential apprehension about differences in treatment provision depending on whether they did/did not participate. Reassurance about that and understanding of their decision appeared to be very important to them.

*"I wouldn’t be one for ill-informed decisions, or just maybe getting pushed into things… So for me, it was a huge part of making the decision." Participant7*

Ability to withdraw from the study at any point or for women to change their mind appeared to be something that resonated with several women. The security of being able to opt out without penalty or ill-effects, at any time, was also viewed as a reassuring aspect of the decision to participate in the trial. Giving time to make the decision is also something that women looked favourably on when deciding to participate.

### Trial considerations

This main theme provides an account of some of the issues surrounding certain aspects of the trial design and conduct that appear to have had an influence on women’s decision-making process to participate in the trial. The subthemes described are mainly centred on the treatments that were available in the trial, trial design and the perceived impact of trial obligations.

Overall, it appears that participants were very well informed and had good basic knowledge of the design of the trial in terms of randomisation, blinding and the use of placebo. A few participants showed an awareness of the need for a placebo group in randomised controlled trials.

*"Well that’s the only way trials work is with placebos so…Because you need to have a controlled group. To know if you’re active [medication], is actually working." Participant9.*

Having a placebo arm did not appear to have an impact on the decision to participate. It also transpired that generally, there was a good level of understanding about the use and importance of blinding in clinical trials with some participants recognising that this would often be a standard aspect of clinical trial.

*"that’s normal practice I think if you’re doing a research trial"Decliner1*
*"Strengthens the trial, doesn’t it, in the long run" Participant4*

Blinding was not a cause for concern with anyone and it was viewed as a very positive aspect as participants felt everyone would be treated the same when nobody knew who is on the medication or placebo. Added security of knowing there was an unblinding procedure, where necessary, also eased women’s minds.

*"It was probably better that nobody knew, because I was getting the same treatment as everyone else in a way" Participant6*

Overall, it appears the trial design was not a significant factor for women when deciding to participate.

All participants talked about the convenience of the study visit times. The study visits coincided with standard care maternity appointments and this appeared to be an important factor for women. Responses from the interviews regarding the timing of the study visits included feelings of being facilitated and flexible in relation to their study visits. Quite a few women also appreciated that the study visits were relatively short and did not inconvenience them too much.

*"I mean it’s very convenient. I can come whenever suits me, so it’s brilliant. It works around my other clinic, so it’s great."Participant6*

Overall, women’s thoughts and feelings were that the trial was well organised. Convenient location, study visit times and the added flexibility around visits were viewed positively and women were generally very happy with the conduct of the trial and its accessibility for them.

## Discussion

Main findings suggest that a significant perception of personal benefit from participation was a big influence on women’s decisions to participate. This mainly centred on the fact that the intervention treatment was viewed more favourably than the current standard of care treatment for GDM. Women also viewed GDM as a significant obstetric condition (and were quite overwhelmed with their diagnosis) and wanted to minimise the adverse effects of the condition on their pregnancy as much as possible by participating in the trial. Overall, the perception of risk in this trial was low, thus, women reported more potential benefits than risks of participating, including the benefit of being able to contribute to medical research to help other pregnant women with GDM in the future.

Aspects of the recruitment process like what, and how, information on the trial was received; frequent and personal interactions with the study team; voluntariness of participation and accessibility of the trial were all viewed as key aspects relating to their decision-making. Interestingly, in this study, the trial design and treatment allocation did not appear to have a significant impact on women’s decisions.

### Personal perspectives and perceived benefits

The findings of this study compare well with other similar studies. Personal benefit from participating in the trial and the idea of a potentially beneficial intervention treatment, has been widely cited as being one of the most influential factors for women to participate in clinical trials during pregnancy ( Drapkin-Lyerly *et al.*, 2012; Kenyon *et al.*, 2006; Oude Rengerink *et al.*, 2015; Smyth *et al.*, 2012). In this study the possibility of receiving a more favourable treatment option to control GDM was certainly a motivating factor for the majority of women.

Fear of the effects of the condition on their pregnancy appeared to be a factor considered by women when making their decision to participate in this trial. Considering the clinical circumstances and women’s perceptions of their own situations is also something that has been recognised in other similar studies as playing a role in how women decide to participate in clinical trials during pregnancy ( Oude Rengerink *et al.*, 2015; Smyth *et al.*, 2012). Drapkin-lyerly *et al.* (2012); Meshaka *et al.* (2016) and Mohanna & Tunna (1999) refer to the idea that women consider the personal relevance of the research in relation to participation, in that, if women perceive being personally affected by the condition or potential condition then they may be more likely to take part. In Drapkin-Lyerly *et al.*’s (2012) study of women’s participation in H1N1 vaccine trials, women appeared more concerned about contracting H1N1 than getting the vaccine and this influenced their participation. Similarly, although reporting on an observational trial, Meshaka *et al.* (2016) reported that fear of being affected by a condition (diabetes) influenced women’ decisions to participate in the trial. Mohanna & Tunna’s (1999) study on decliners also refers to this theme of research relevance and reported that when women did not perceive the research relevant to them, they declined participation.

The importance of contributing to research and being able to help other pregnant women in the future, as also recognised in studies by Ballantyne *et al.*, 2017; Drapkin-Lyerly *et al.*, 2012; Oude Rengerink *et al.*, 2015 and Kenyon *et al.*, 2006) appeared to be a significant motivating factor for women participating in EMERGE. Some women reported participating purely for helping medial research and others spoke about helping other pregnant women in the future, but also mentioned some potential personal gain if they were to develop GDM in subsequent pregnancies. Conditional altruism, also recognised elsewhere ( Kenyon *et al.*, 2006), was somewhat evident in this study as generally the perception of risk was low, there was perceived personal gain and some women mentioned that it was no inconvenience for them to participate and therefore, why not help research.

### Perception of risk

Women’s perceptions of risks associated with participation in clinical trials appear to play a large role in decision-making. Feelings of no risk with the trial appeared to make the decision to participate easier for women in studies by Ballantyne *et al.* (2017), a trial of a probiotic in pregnancy, and Kenyon *et al.* (2006), a trial of antibiotics in pre-term labour. In Drapkin-Lyerly *et al.*’s (2012) study the perception of risk was focused more on a fear of contracting the H1N1 flu than getting the vaccine and therefore influenced participation.

In this study, the general perception of risk was low. Women were aware that the intervention medication had been researched in pregnancy previously and is used in some countries as standard of care treatment for GDM. The fact that it was not a completely new experimental treatment rendered this trial low risk for most women. If the treatment was a new medication being tried out in pregnancy the response may have been different and this was mentioned by some women. Similar attitudes were also expressed by women in a study by Drapkin-lyerly *et al.* (2012).

### Recruitment process and the role of others

The quality and means of providing information about trials has been widely cited as pivotal when making decisions to participate in clinical trials ( Baker *et al.*, 2005; Kenyon *et al.*, 2006; Locock & Smith, 2011; Mohanna & Tunna, 1999; Oude Rengerink *et al.*, 2015; Salazar *et al.*, 2016; Smyth *et al.,* 2012; Tooher *et al.*, 2008). Having clear, understandable and detailed written information was cited as important, however, what appeared to be more significant was the personal interaction, with healthcare professionals and the study team, to further explain the trial to potential participants ( Baker *et al.*, 2005; Kenyon *et al.*, 2006; Locock & Smith, 2011; Mohanna & Tunna, 1999; Oude Rengerink *et al.*, 2015; Tooher *et al.*, 2008). This was also evident in this study where receiving detailed information about the study and having the opportunity to seek further information from the study team appeared very influential in women’s decision-making process.

Voluntariness of participation resonated very much with the women in this study. Tooher *et al.* (2008) recognised that women may feel coerced into participation, especially if they believe care would be compromised if they refuse consent. Knowing that care would not be compromised and not feeling coerced into participating was appreciated and expressed by most women in this study. The importance of this aspect of recruitment has also been reported in other studies in terms of influences on participation ( Kenyon *et al.*, 2006; Smyth *et al.*, 2012). Most women reported that they discussed the trial with their partners, however, several reported that their decision to participate was personal. The role of partners in decision making for participation in pregnancy research has been explored, resulting in mixed responses about their influence ( Baker *et al.*, 2005; Ballantyne *et al.*, 2017; Ngure *et al.*, 2017; Smyth *et al.*, 2012). Baker *et al.* (2005) suggested that involvement of partners in decision making differed for women depending on the potential impact on the baby. When there was no perceived impact on the baby women were more likely to make an autonomous decision ( Baker *et al.*, 2005). Given the perception of low risk in this study, this may have played a role in why many of the decisions appeared to be made personally by women to participate in this trial.

When it comes to the recruitment process, it appears that receiving detailed information and personal interactions with trusted clinicians and the study team are hugely important and have a considerable impact on women’s decision-making process to participate in clinical trials. In addition, testimonials from previous participants on their experiences was also a significant suggestion from women on how their decision-making process could be enhanced.

### Trial conduct and design

Women’s opinions and responses to the trial design in this study are very interesting in comparison to other similar studies. Women in this study appeared to have a good understanding of the trial design and were quite knowledgeable about the use of placebo, blinding and randomisation in clinical trials. The randomised double-blind placebo-controlled trial design of EMERGE did not seem to have a significant impact on the decision.

Studies by Ballantyne *et al.* (2017); Drapkin-Lyerly *et al.* (2012); Kenyon *et al.* (2006); Tooher *et al.* (2008) and Mohanna & Tunna (1999) all reported negative opinions from participants about the use of placebos in clinical trials, with participants more likely to decline participation in placebo-controlled trials or perceived themselves to be at a disadvantage if randomised to the placebo arm. Findings from this study appear different. A small number of women did express a preference for the active treatment, but overall did not mind which treatment arm they were allocated to and reported that it was not something that they appeared to focus on too much when making their decision.

Randomisation is also an aspect of trial designs that appears to be disliked in general. A few studies report participants having a preference for non-randomised trials ( Baker *et al.*, 2005; Oude Rengerink *et al.*, 2015; Tooher *et al.*, 2008). While most women in this study admitted not knowing much about the detailed concept of randomisation in clinical trials, some could provide a basic explanation and assumed it to be a normal aspect of conducting trials, thus, were not concerned either way.

Responses on the blinding of the trial were very interesting in this study. Other studies have reported preferences for unblinded trials and concerns regarding blinding ( Kenyon *et al.*, 2006; Tooher *et al.*, 2008), whereas in this study the blinding aspect was viewed very favourably. It was considered important that women were not treated differently depending on what treatment they were allocated and a protection against this was that everyone was blinded to the allocations. Trust in the study team and knowledge that there was an unblinding procedure in place, if needed, made women more comfortable with blinding.

Practical considerations of participating were important. Women reported that study visits that coincided with their antenatal clinic visits were very convenient and this had some influence on their participation. Some women admitted that if the study visits weren’t coinciding with standard care it would have been very difficult to participate. Trial inconvenience and time commitments was also a considerable issue reported by women in studies by Ballantyne *et al.* (2017) and Baker *et al.* (2005). Planning study visits around standard care visits was highlighted by Salazar *et al.* (2016) as a means of improving recruitment and retention in clinical trials, and evidence from this study suggests it is an important aspect of trial conduct to consider when planning clinical trials in pregnancy.

### Differences in perceptions between participants and decliners

It was difficult to make a comprehensive comparison between participants and decliners in this study due to the small sample size of decliners. However, from the sample that participated in this study some insight was obtained. Perceived benefits to participation appeared to differ between the groups with all the participants of EMERGE reporting a potential personal benefit to participation, whether it was their primary influencing factor or not, whereas the decliners did not appear to perceive any personal benefits to participation.

General responses to their diagnosis of GDM was similar in both groups where there was evidence of apprehension, upset and a sense of being overwhelmed by the diagnosis and information provided on GDM. However, how they perceived the research relevance to them appeared to be slightly different. It appeared that the decliners expressed thoughts that they may not require medication to treat GDM in their pregnancies. This perception has also been cited in other studies where women who did not perceive the research relevant to them declined participation ( Mohanna & Tunna, 1999). The participants of EMERGE did not necessarily indicate that they thought they would need insulin, however, by participating in the trial their perspectives were that they were reducing the chance of this occurring. 

Perception of risk appeared to be higher in the decliner group where fear of unknown effects on the baby was the primary reason for declining participation in one case. The importance of having personal contact with study team members and the opportunity to have a thorough discussion about the study was highlighted by both participants and decliners indicating the pivotal role this aspect of recruitment plays in decision-making.

Opinions on the trial design were similar. Both participants and decliners appeared to have similar knowledge about the design of the trial and did not have significant opinions on it in relation to their decision to participate or decline. This contrasts with other studies which found that decliners were less inclined to participate in trials if there was a placebo arm ( Mohanna & Tunna, 1999), or that being in the placebo arm would be perceived as a disadvantage ( Ballantyne *et al.*, 2017). Neither of the decliners spoke about a disadvantage with placebo, blinding or randomisation aspects of the trial. Overall, it appears participants perceived more advantages and benefits to participation whereas decliners appeared to focus more on the potential risks and perceived burdens of participating in the trial.

### Limitations

This study had a number of limitations. The main limitation in this study was the small sample size of decliners of EMERGE. It was difficult to obtain decliners for this study. Although the findings from the two decliners provided important insight into decliners perspectives on participation in EMERGE it is not representative of all decliners, thus, findings from decliners perspectives should be interpreted carefully.

 Another limitation was the relatively restricted variation in participant characteristics. A maximum demographic variation sampling technique was employed during recruitment; however, it was difficult to obtain a wide variation of characteristics. Methodological challenges such as the potential for EMERGE participant bias when answering questions during interview, due to their pre-existing relationship with the researcher for this study, and potential researcher bias, due to their concurrent involvement in the EMERGE clinical trial, were also potential limitations of this study. This was recognised and acknowledged prior to starting the study and actions were taken to minimise their effect during the study by consulting and peer debriefing with an independent qualitative researcher throughout the planning and data analysis stages.

## Conclusion

Conducting randomised controlled trials of medication in pregnancy are essential to produce an evidence base to appropriately address the health needs, and improve healthcare, for this population. Endicott & Haas (2012) have recognised that there appears to be an increase in the conduct of therapeutic drug trials during pregnancy. This is encouraging, however, recruitment of pregnant women into clinical trials remains challenging for researchers and recruitment rates appear to be consistently low in pregnancy trials ( Strömmer *et al.*, 2018). Overall, women viewed participation in this clinical trial very positively and this study provided an insight into how pregnant women with GDM view clinical trials and their main motivating factors for participation. Some insight was also gained into why decliners decided not to participate in EMERGE, and perhaps what they considered important when invited to participate in the trial.

### Recommendations for practice/future research

Findings from this study reveal important recommendations for enhancing recruitment into randomised controlled trials of medications during pregnancy. Trial information needs to be clear and thorough with appropriate details about the trial, trial design and the intervention medications. Personal contact, availability of trial staff and adequate trial discussion time are essential components of the recruitment process. Testimonials from previous participants are a useful way to provide insight for others into the experiences of pregnant women participating in clinical trials. Voluntariness of participation and withdrawal options should be emphasised. Participants should be facilitated by planning study visits around standard care appointments as much as possible. Study tasks and visit times should be kept to minimum requirements to minimise the inconvenience of the trial for women.

As there was a limited number of decliners of EMERGE included in this study further research on this cohort in the future is needed. This study provided insight into some of the potential reasons for declining participation and considerations of what is important for women during the recruitment process, however, it cannot be representative of all decliners. Exploring the perspectives of a greater number of decliners would add further insight into why women with GDM do not participate in an IMP clinical trial during their pregnancy. This information would be beneficial for researchers to understand the views and concerns of these women which could assist in the future when devising clinical trial designs and recruitment strategies.

There is a growing body of research across many trial types and designs, exploring the reasons for accepting and declining trial participation. Qualitative research has contributed to this body of research. A recent Cochrane qualitative evidence synthesis highlights the importance of person-centred approaches to recruitment that consider the multifaceted decision-making process for those invited to take part in a trial ( Houghton *et al.*, 2020). There is now the need to develop and test appropriate participant focused recruitment interventions, particularly in pregnancy trials.

## Data availability

### Underlying data

Original data cannot be shared as consent was not obtained from participants for secondary use of data, and the transcripts cannot be fully deidentified. Anonymised transcripts may be reviewed upon request to sinead.wallace@live.com but not for reuse in other projects.

### Extended data

Open science framework: Participation in an RCT of metformin in gestational diabetes mellitus (GDM): pregnant women’s perceptions and experiences of the decision-making process. https://doi.org/10.17605/OSF.IO/T7GR8 ( Wallace, 2021).

This project contains the following extended data:

- Participation in an RCT of metformin in gestational diabetes mellitus- interview guide.pdf
- Participation in an RCT of metformin in gestational diabetes mellitus - supplementary data files.pdf

Data are available under the terms of the Creative Commons Zero "No rights reserved" data waiver (CC0 1.0 Public domain dedication).
