# Supplementary material for: Participation in a randomised controlled trial (RCT) of metformin in gestational diabetes mellitus (GDM): pregnant women’s perceptions and experiences of the decision-making process
Source: HRB Open Res. 2021 Jun 14;4:65. [Version 1] doi: 10.12688/hrbopenres.13289.1 (PMC9751497; doi:10.12688/hrbopenres.13289.1)
Supplement: Peer review history

# Peer review: Reviewer response for version 1

**DOI:** 10.21956/hrbopenres.14464.r33081

**Published:** 2022-12-14

**Authors:** Karoline K. Nielsen

**Affiliations:** 1 Health Promotion Research, Copenhagen University Hospital, Steno Diabetes Center, Copenhagen, Denmark

Thank you for the invitation to review this interesting article, which explores pregnant women’s perspectives and experiences in the decision-making process to participate in a RCT of metformin treatment for women with gestational diabetes mellitus (GDM). I would like to applaud the authors for conducting this study of an aspect of RCTs which is often not prioritised, but extremely important and relevant for other researchers planning RCTs among pregnant women in the future. It is also well-written and provides some very useful recommendations for future research and practice.  

 In the following, I present some aspects which could be elaborated or clarified in the paper. I fully acknowledge that, e.g., recruitment can be challenging and that some of the points/aspects may not be possible to address at this stage.

**Abstract:**

 Would be helpful to the reader if the number of themes and subthemes are presented in the abstract.

**Objectives:**

 The third objective regarding exploration of differences between those who decline participation in the EMERGE trial and those who agree, seems to be addressed in the discussion rather than in the results section. This should be revised, so that if presented as a separate objective it should be covered in the results. The current text in the discussion section reads very much like something belonging to the results section. Another option is to remove it as a specific objective and then amend the text in discussion.

**Methods:**

 In general, I find that the methods section is nicely written and for most parts cover the essential aspects required in a qualitative study. However, there are some aspects which could benefit from clarification / elaboration to further enhance the quality of the study.

**Participants and setting:**

 The authors provide a very good explanation for why pre-determined sample sizes are not relevant in qualitative research. However, as a reader I am still not sure how the authors ended up with 11 participants. Was the recruitment and analysis done iteratively, so that it was due to theoretical saturation? Or was it a practical matter? This is particularly relevant regarding the decliners in the EMERGE trial where only 2 were recruited. N = 2 is low, even for qualitative research, and the authors also discuss this in the discussion. However, the reasoning or circumstances for ending up with this sample size could be made clearer in the methods.

 Also, regarding recruitment into this qualitative study, would be useful to know how many EMERGE participants and decliners you approached and invited to participate in the qualitative study. Did anyone you approach, say no to participate in an interview? And if so, did they give a reason?

 What information were potential participants in the qualitative study given about the aim/purpose of the qualitative study?

 Where did the interviews take place? Eg in a clinical setting or in a private setting?

 How long had the participants been enrolled in the EMERGE trial at the time of the interviews?

**Data collection:**

 The authors state that the interview guide was developed from a review of previous literature. Would be helpful for the reader, if examples of what the questions or themes in the interview guide were provided.

**Rigor:**

 The authors provide some nice considerations regarding social desirability bias, which I agree is very relevant to the study. However, would be good if the authors could elaborate on the background of SW as the one conducting and analysing the interviews. Also, the influence of the researchers and their role in EMERGE may not only have influenced what was said by the participants, but also what was given attention to in the analysis. This should be acknowledged as well here and in the discussion.

**Results:**

 Overall, the authors provide a clear and well-written description of the different themes. For the first theme (page 6 of the pdf) where there is a description about risk perception, it would be helpful to have some more information on what participants in the EMERGE trial were told about the risks/potential side effects of participating. To better understand the context.

 Second theme (page 7), the authors note that women reported that they trusted their HCP to prioritise their best interest and not unnecessarily put them at risk. This seems to be linked to theme 1 and the part about risk perceptions?   

**Discussion:**

 The part of the discussion where the authors discuss their findings in relation to other studies feels perhaps a bit long and repetitive, but this is a very minor comment.

 As mentioned earlier, the section on the differences between participants and decliners need some revision. I would also advise the authors to be more cautious about what can be concluded for this part considering there was only 2 decliners.

 In the limitations, it would make the arguments stronger and be very helpful to other readers if the authors elaborate on why it was 1) difficult to recruit decliners and 2) difficult to obtain wide variation of characteristics. Also, what implications do this have for the findings?

**Recommendations for practice/future research:**

 Generally, a nice and useful section. However, I would suggest that the sentence “however, it cannot be representative of all decliners” is changed. First, findings from qualitative research will rarely be representative of all. Second, with only 2 decliners in your study, I think it would be more reasonable to say that you only provide some indications of what may be the perceptions and experiences of decliners, and that a more thorough exploration of this group and how they differ from those agreeing to participate is still needed.   

**Minor:**

 I will advise the authors to refrain from using the word “significant” or “significantly” e.g. “have significantly influenced their decision”. Simply because researchers with very limited understanding of qualitative research sometimes misunderstand/misinterpret the use of the word when presented in qualitative research.

Is the work clearly and accurately presented and does it cite the current literature?

Yes

If applicable, is the statistical analysis and its interpretation appropriate?

Not applicable

Are all the source data underlying the results available to ensure full reproducibility?

Yes

Is the study design appropriate and is the work technically sound?

Yes

Are the conclusions drawn adequately supported by the results?

Yes

Are sufficient details of methods and analysis provided to allow replication by others?

Yes

Reviewer Expertise:

Gestational diabetes, public health, interventions

I confirm that I have read this submission and believe that I have an appropriate level of expertise to confirm that it is of an acceptable scientific standard.

# Peer review: Reviewer response for version 1

**DOI:** 10.21956/hrbopenres.14464.r30310

**Published:** 2021-10-20

**Authors:** Luke E. Grzeskowiak

**Affiliations:** 1 SAHMRI Women and Kids, South Australian Health and Medical Research Institute, Adelaide, SA, Australia; 2 College of Medicine and Public Health, Flinders University, Adelaide, SA, Australia

Thank you for the opportunity to review this study investigating women’s perceptions and experiences of the decision-making process of participating in a clinical trial involving the use of metformin for the treatment of gestational diabetes. This is an important and interesting study that has broad applications to research involving pregnant women. The article is clear and well written and I enjoyed reading it. I have no doubts that this is worthy of indexing.

 The research questions are clearly described and the description of the methods are sound. I have paid particular attention to the reported methods in comparison to the COREQ guidelines for reporting qualitative studies and could only identify a few aspects that would benefit from further clarification:

1. Credentials and experience/training of the researcher conducting the interviews.
2. Any information available regarding non-participation in interviews? How many were approached in order to consent 11?
3. Were field notes collected during interviews?
4. What was the duration of the interviews?
5. Were transcripts returned to participants for comment/correction?
6. Did participants provide feedback on the findings?

There is a little bit of confusion caused by the use of the term participants in the manuscript which in some cases refers to participants of EMERGE and in other cases refers to participants of this qualitative study. A minor issue but I wonder if clarity could be improved by use of alternative terms. I do note in the methods that the term acceptors and decliners was used, rather than participants and decliners.

 I would have liked to have seen more emphasis on better understanding why women decline participation, but I understand this is a difficult group to recruit. This limitation is acknowledged and I respect the authors candour in stating the results cannot be representative of all decliners. I do. However, wonder if any negative aspects could have been highlighted in the results. There are only two quotes from decliners and they don’t appear to relate to anything that would have led them to decline. In the discussion we are introduced to a couple of concepts potential related to declined participation (i.e. perceived high risk), but this doesn’t appear to have been introduced in the results earlier.

Is the work clearly and accurately presented and does it cite the current literature?

Yes

If applicable, is the statistical analysis and its interpretation appropriate?

Not applicable

Are all the source data underlying the results available to ensure full reproducibility?

Yes

Is the study design appropriate and is the work technically sound?

Yes

Are the conclusions drawn adequately supported by the results?

Yes

Are sufficient details of methods and analysis provided to allow replication by others?

Yes

Reviewer Expertise:

Pharmacy practice; Obstetric and neonatal pharmacology

I confirm that I have read this submission and believe that I have an appropriate level of expertise to confirm that it is of an acceptable scientific standard.
